# Correction: Reproducible big data science: A case study in continuous FAIRness

**DOI:** 10.1371/journal.pone.0294883

**Published:** 2023-11-21

**Authors:** Ravi Madduri, Kyle Chard, Mike D’Arcy, Segun C. Jung, Alexis Rodriguez, Dinanath Sulakhe, Eric Deutsch, Cory Funk, Ben Heavner, Matthew Richards, Paul Shannon, Gustavo Glusman, Nathan Price, Carl Kesselman, Ian Foster

The URLs in the Data Availability statement for this paper are incorrect. The correct URLs are:

- DNase-Seq has the unique identifier minid:b9dt2t and is available from https://identifiers.org/minid:b9dt2t
- Aligned BAM files have an identifier: minid:b9vx04 and are available from https://identifiers.org/minid:b9vx04
- The collection of BED files of footprints have an identifier: minid:b9496p and are available from https://identifiers.org/minid:b9496p
- The non-redundant Motifs database has an identifier: minid:b97957 and is available from https://identifiers.org/minid:b97957
- The motif intersected hits have an identifier: minid:b9p09p and are available from https://identifiers.org/minid:b9p09p
- The Transcription Factor Binding Sites generated from the study have an identifier: minid:b9v398 and are available from https://identifiers.org/minid:b9v398

## References

[1] Madduri R, Chard K, D’Arcy M, Jung SC, Rodriguez A, Sulakhe D, et al. (2019) Reproducible big data science: A case study in continuous FAIRness. PloS ONE 14(4): e0213013. 10.1371/journal.pone.0213013 30973881 PMC6459504

